# Advanced glycation end products are associated with limited range of motion of the shoulder joint in patients with rotator cuff tears associated with diabetes mellitus

**DOI:** 10.1186/s12891-022-05229-5

**Published:** 2022-03-22

**Authors:** Issei Shinohara, Yutaka Mifune, Atsuyuki Inui, Hanako Nishimoto, Kohei Yamaura, Shintaro Mukohara, Tomoya Yoshikawa, Tatsuo Kato, Takahiro Furukawa, Yuichi Hoshino, Takehiko Matsushita, Ryosuke Kuroda

**Affiliations:** Department of Orthopaedic Surgery, Kobe University Graduate School of Medicine, 5-2, Kusunoki-cho7, Chuo-ku, Kobe-shi, Hyogo 650–0017 Japan

**Keywords:** Advanced glycation end products, Shoulder capsule, Rotator cuff tears, Range of motion, Reactive oxygen species, Cell viability

## Abstract

**Background:**

Most degenerative rotator cuff tears (RCTs) are associated with a limited range of motion (ROM) of the shoulder joint. Additionally, patients with diabetes mellitus (DM) show a higher frequency of limited ROM. Recently, advanced glycation end products (AGEs) of proteins have been observed to cause tissue fibrosis, primarily through abnormal collagen cross-linking and oxidative stress. In this study, we investigated the effect of AGEs on ROM limitation in the shoulder capsule and its relationship with DM in the patients with RCTs.

**Methods:**

Sixteen patients (eight in the DM and non-DM groups) who underwent arthroscopic surgery for RCT with limited shoulder ROM were included in this study. AGE-related pathologies in both groups were compared, and the relationship between AGE accumulation and shoulder joint ROM was evaluated. Shoulder capsule tissue was harvested and subjected to histological and in vitro evaluation.

**Results:**

The DM group displayed high levels of AGEs and reactive oxygen species (ROS), and reduced cell viability. There was a significant positive correlation between ROS expression, apoptosis, and preoperative hemoglobin A1c. ROS expression, apoptosis, and ROM of the shoulder joint showed a negative correlation. The NADPH oxidase (NOX) expression and collagen III/I ratio were significantly higher in the DM group than in the non-DM group.

**Conclusions:**

The DM group showed significant AGEs deposition in the shoulder capsule. Additionally, there was a significant association between AGEs and ROM limitation. Collectively, the findings suggest that the oxidative stress induced by AGEs deposition, which leads to fibrosis and local inflammation, might contribute to the limited ROM of the shoulder joint in patients with RCTs accompanied by DM.

## Background

Passive range of motion (ROM) limitation of the shoulder is common in patients with rotator cuff tears (RCTs), with more than 40% of patients with full-thickness tears showing mild or moderate limitation [1]. The pathophysiology of limited ROM of the shoulder joint after RCTs has been reported to be associated with synovial inflammation [2] and contracture of the soft tissues surrounding the glenohumeral joint (secondary frozen shoulder) [3, 4]. Histologically, fibroblast proliferation into the type I collagen matrix of the shoulder capsule has been reported [5]. Fibroblast proliferation causes capsular fibrosis and contracture, which is considered a mechanism of limited ROM [6]. However, the etiological mechanism of fibroblast proliferation into the shoulder capsule and limited ROM of the shoulder joint after RCT remain unclear.

Advanced glycation end products (AGEs), the final glycation products of proteins, are formed by a non-enzymatic reaction between the ketone group of reducing sugars and the free amino group of proteins, resulting in progressive rearrangement, dehydration, and condensation [7]. Owing to their slow metabolism, AGEs tend to accumulate and cause the abnormal cross-linking of collagen, which leads to inflammation and tissue fibrosis [8]. Furthermore, it has been shown that AGEs cause hand and shoulder disability [9]. Previous studies on patients with frozen shoulder, shoulder instability, and RCTs demonstrated that AGE deposition in the shoulder capsule was significantly higher in patients with frozen shoulder than in healthy subjects [6]. In addition, AGEs produce reactive oxygen species (ROS) via NADPH oxidase (NOX) by binding to the receptor of AGEs (RAGE) [10]. ROS generation induces oxidative stress, which in addition to the abnormal cross-linking of collagen, causes tissue damage and fibrosis [8, 10].

The accumulation of AGEs is associated with the duration of hyperglycemia [11]. Aging and diabetes mellitus (DM) are reported risks for accumilation of AGEs [12, 13]. DM is also a risk factor for shoulder contractures [14, 15] and some reports suggest an association between AGEs and contractures [6]; indeed, the frequency of shoulder contractures is four to five times higher in patients with DM than in those without DM [16]. Hence, we hypothesized that the accumulation of AGEs in the shoulder capsule of DM patients leads to limited ROM after RCTs. To test this hypothesis, this study was aimed to investigate the effect of AGEs on ROM after RCTs in patients with DM by comparing the ROM of the shoulder joint with and without DM and the pathology associated with AGEs.

## Methods

### Isolation of samples from the shoulder capsule

The Ethics Committee of our institute approved this study and informed consent was obtained from all the patients involved. Patients with limited ROM of the shoulder joint associated with degenerative RCT were included in the study (exclusion criteria: traumatic RCTs, rotator cuff retear, frozen shoulder without RCT, calcific tendinopathy, osteoarthritis, and rheumatoid arthritis). To conform to ethical standards, we did not collect shoulder capsule samples from patients with RCT who did not have limited ROM of the shoulder joint.

Sixteen patients (average age of 65.9 years; nine men and seven women), who underwent surgical treatment for RCT with limited ROM of the shoulder joint at our hospital over a two-year period starting in January 2019, were chosen for the study. The passive ROM of the shoulder joint was measured under general anesthesia at the time of surgery. Patients with limited ROM and manipulation compared to the healthy side with normal findings on radiographs (excluding arthritic changes in the glenohumeral joint and calcific tendinopathy) were included in this study. To evaluate the association of AGEs with pain associated with RCTs and ROM limitation due to immobilization, patients with adhesive shoulder arthritis without RCTs were excluded from the study. For ethical reasons, patients with no ROM limitation were also excluded. Finally, eight cases with DM (DM group) and eight without DM (control group) were evaluated in this study. The DM group was defined as those with a preoperative HbA1c level of ≥6.5 [17]. The sample size was determined by power analysis based on data from the previous study using G*Power 3.1. A shoulder ROM of 40° may be sufficient to have clinical significance according to a similar study [6]. Preliminary sample size calculations indicated that a difference in shoulder ROM (anterior elevation: AE) of 40° in the two groups was detectable with a sample size of 16 participants (8 in each group) using a t-test (effect size = 1.33, α = 0.05, power = 0.81). Furthermore, a post-hoc calculation of the sample size using the results of this study yielded a power of 0.97 (effective size = 1.9, α = 0.05).

Tissue from the shoulder capsule was obtained intraoperatively, and the tissue specimens were excised by the same surgeon (Y. M.). The shoulder capsule tissue was harvested using basket forceps from a site medial to the subscapularis tendon and adjacent to the joint labrum, as previously described [6]. A portion of the harvested shoulder capsule tissues was fixed in formalin for tissue staining, and the remaining tissues were minced to a size of approximately 1 mm^3^ and cultured. The minced tissue was cultured in a monolayer on a 100 mm-diameter culture dish in Dulbecco’s modified Eagle’s medium (DMEM, HyClone, Logan, UT, USA) mixed with 10% fetal bovine serum (FBS, Cansera, Rexdale, Ontario, Canada), 100 U/mL penicillin, and 100 μg/mL streptomycin. Cultures were incubated at 37 °C in a humidified atmosphere of 5% CO_2_/95% air and passaged for 1–2 weeks. Cells in passages 2 and 3 were used in this study.

### Histological examination

**Fig. 1:**
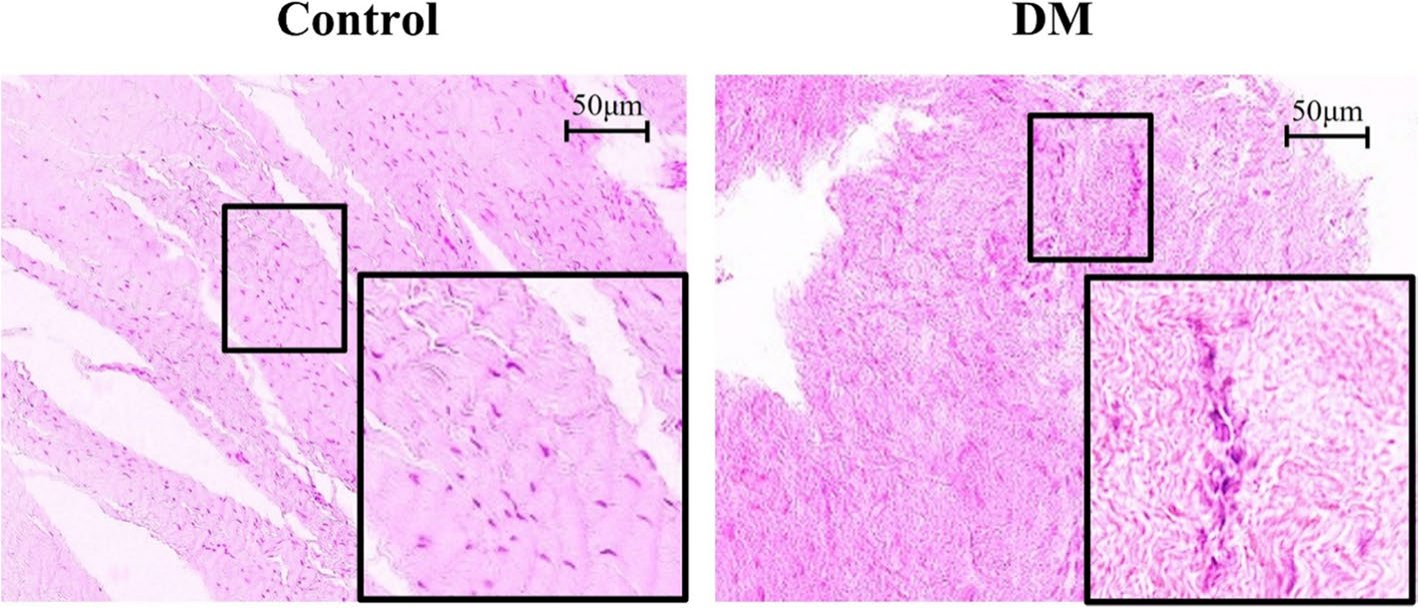
Hematoxylin and eosin (H&E) staining of the shoulder capsule cells shows dense fibroblasts and disorganized collagen arrangement in the DM group

**Fig. 2:**
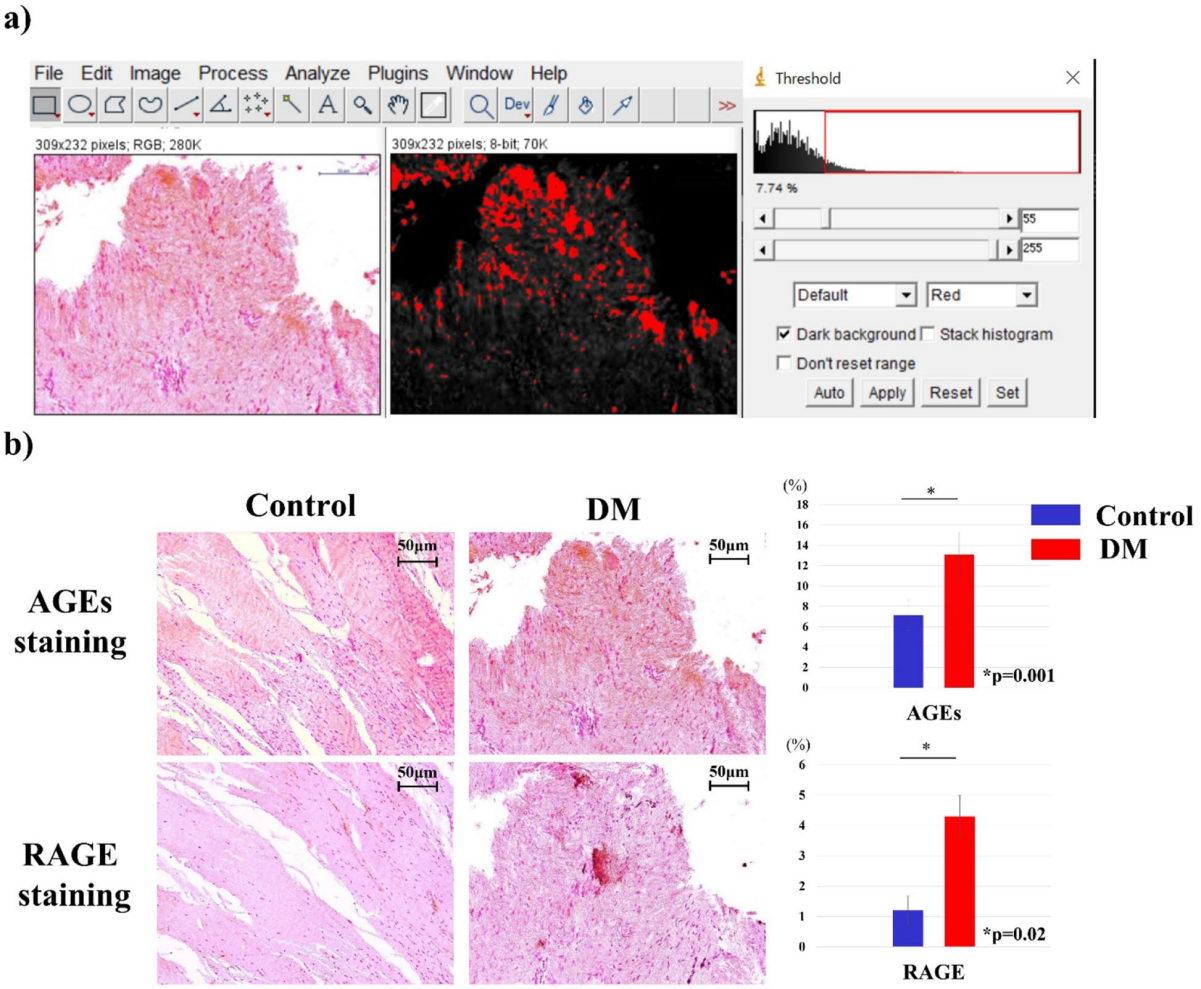
Immunostaining for advanced glycation end products (AGEs) and receptor of AGEs (RAGE). **a** Quantification of the percentage of immunostaining to H&E staining using ImageJ. **b** Both AGEs and RAGE staining were significantly higher in the DM group (*p* < 0.05) than in the control group

After 24 h, formalin-fixed tissues were dehydrated and embedded in paraffin wax. Five micrometer sections were mounted on slides. Hematoxylin and eosin (H&E) staining (Fig. 1) and immunostaining were performed (Fig. 2). For immunostaining, the cells were deparaffinized, dehydrated, and incubated overnight at 4 °C with the primary antibodies anti-AGE (10 μg/mL, Abcam, Cambridge, UK) and anti-RAGE (10 μg/mL, Abcam). After overnight incubation, the sections were incubated with secondary antibodies (Histofine Simple Stain MAX Po; Nichirei Bioscience, Tokyo, Japan) at room temperature (25 °C) for 1 h and counterstained with hematoxylin. Digital images of the slides were taken using BioZero BZ-8000 (Keyence, Osaka, Japan). The color deconvolution plugin of Image J (ver. 1.52), a public domain Java-based image processing software developed by the National Institutes of Health (NIH), was used to quantify the stained areas [18]. In brief, the color images were digitally separated into red, green, and blue images. Pixel subtraction was performed from the red image to the blue image; thereafter, the average pixel intensity of the subtracted image was calculated (Fig. 2a). The percentages of the AGEs and RAGE staining were calculated as the average of the four fields of view.

### Cell viability (cell proliferation assay)

Cell viability was measured with the water-soluble tetrazolium salt (WST) assay using the Cell Counting Kit-8 (Dojindo, Kumamoto, Japan), and 5000 cells were seeded in each of the 96 wells and cultured in DMEM for 24 h. After incubation, 10 μL of WST was added to each well and incubated at 37 °C for 4 h, after which soluble formazan was quantified in viable cells. Cell viability was evaluated by measuring the absorbance of the reduced formazan at 450 nm using the WST assay.

### ROS expression

Fluorescence immunostaining was used to assess the effect of ROS on cells derived from the shoulder capsule. Based on previous reports [19], the ROS expression was detected using the Total ROS/Superoxide Detection Kit (Enzo Life Science, Farmingdale, NY, USA). Cells (5 × 10^4^ cells/mL) were incubated with the oxidation-sensitive fluorescent probe dichloro-dihydro-fluorescein diacetate (DCFH-DA) at a final concentration of 10 μM for 60 min in the dark at 37 °C. After incubation, the cells were washed with PBS and resuspended in trypsin. For quantification, the number of ROS-positive cells and diamidino-2-phenylindole (DAPI)-positive cells in the four fields of view (0.75 mm × 1.0 mm) of each slide were counted and the mean value was calculated.

### Apoptosis rate

Two days after incubation, immunofluorescence staining was performed to compare the apoptosis rates of the cells. The rate of apoptosis was determined by terminal deoxynucleotidyl transferase dUTP nick end-labeling (TUNEL) staining using the APO-DIRECT kit (Phoenix Flow Systems, San Diego, CA, USA) according to the manufacturer’s protocol. The ratio of green-stained nuclear fragments to DAPI-stained cells was calculated for each of the four fields of view.

### Quantitative real-time PCR

Cells were seeded in 12-well culture plates at a density of 1.0 × 10^5^ cells/well and cultured in DMEM for 48 h. Total RNA was extracted using the RNeasy Mini Kit (Qiagen, Valencia, CA) according to the manufacturer’s protocol. Oligo (deoxythymidine)-primed first-strand cDNA was synthesized using the High Capacity cDNA Transcription Kit (Applied Biosystems, Foster City, CA, USA). Quantitative real-time PCR was performed in a 20 mL reaction mixture using the SYBR Green Master Mix reagent (Applied Biosystems) on an ABI Prism 7500 Sequence Detection System (Applied Biosystems). The PCR conditions were as follows: one cycle at 95 °C for 10 min, followed by 40 cycles at 95 °C for 15 s, and 40 cycles at 60 °C for 1 min.

The messenger RNA (mRNA) levels of type I collagen (COL1), type III collagen (COL3), RAGE, NOX1, NOX4, interleukin-6 (IL-6), and IL-1β were evaluated using the method described above. The primer sequences are listed in Table 1.

**Table 1:** Primers for Real-time PCR

Gene	Forward Primer (5′ to 3′)	Reverse Primer (5′ to 3′)
NOX1	GGTTTTACCGCTCCCAGCAGAA	CTTCCATGCTGAAGCCACGCTT
NOX4	GCCAGAGTATCACTACCTCCAC	CTCGGAGGTAAGCCAAGAGTGT
RAGE	CACCTTCTCCTGTAGCTTCAGC	AGGAGCTACTGCTCCACCTTCT
COL1	AGGAATTCGGCTTCGACGTT	GGTTCAGTTTGGGTTGCTTG
COL3	GGGAACAACTTGATGGTGCT	CCTCCTTCAACAGCTTCCTG
Nox4	AGTCAAACAGATGGGATA	TGTCCCATATGAGTTGTT
IL6	AGACAGCCACTCACCTCTTCAG	TTCTGCCAGTGCCTCTTTGCTG
IL1β	TACGAATCTCCGACCACCACTACAG	TGGAGGTGGAGAGCTTTCAGTTCATATG
GAPDH	GTCTCCTCTGACTTCAACAGCG	ACCACCCTGTTGCTGTAGCCAA

Primers used in this study

Relative gene expression levels were calculated using the DD-Ct method, with GAPDH as a reference. The expression of each gene was compared between the control and DM groups.

### Statistical analysis

All data are expressed as the mean ± standard deviation. Cell viability and real-time PCR results were expressed as n-fold differences from the baseline control at the corresponding time point. Mann–Whitney’s U test was performed to compare the control group with the DM group. The relationship between the amount of AGEs and ROM was evaluated using Pearson’s correlation coefficient. Results with a *p*-value < 0.05 were considered significant. The data were analyzed using SPSS v23.0 (IBM Corporation, Armonk, NY, USA).

## Results

### Patient background

The DM group consisted of four men and four women with a mean age of 64.0 years. The control group consisted of five men and three women with a mean age of 65.5 years. There was no significant difference in the average age between the two groups. In the DM group, the mean preoperative HbA1c was 7.3 ± 1.2 (6.5–9.7). The mean preoperative HbA1c level in the control group was 5.9 ± 0.2 (5.6–6.3). The mean preoperative passive ROM of the shoulder joint was 126.4 ± 36.3° of AE and 36.4 ± 6.9° of external rotation (ER) in the control group. In contrast, in the DM group, the mean ROM of AE was 90.0 ± 8.9° and that of the ER was 26.4 ± 11.2°. The ROM for both AE and ER was significantly greater in the control group than in the DM group (*p* = 0.030 for AE, *p* = 0.002 for ER).

### Histology and immunohistochemistry

H&E staining showed dense fibroblasts in the DM group. The fiber structure and arrangement of collagen were disorganized in the DM group, whereas they had an almost parallel orientation in the control group (Fig. 1). Immunostaining for AGEs and RAGE predominantly stained the ECM and perinuclear region of the cell, respectively. In the quantification by Image J (Fig. 2a), the average percentage of AGE staining was 7.15 ± 0.65% and 13.1 ± 2.1% in the control and DM groups, respectively, and was significantly higher in the DM group (*p* = 0.001, Fig. 2b). The percentage of RAGE staining was 1.2 ± 0.56% and 4.3 ± 0.87% in the control and DM groups, respectively, and was significantly higher in the DM group (*p* = 0.02, Fig. 2b).

### Cell viability (cell proliferation assay)

**Fig. 3:**
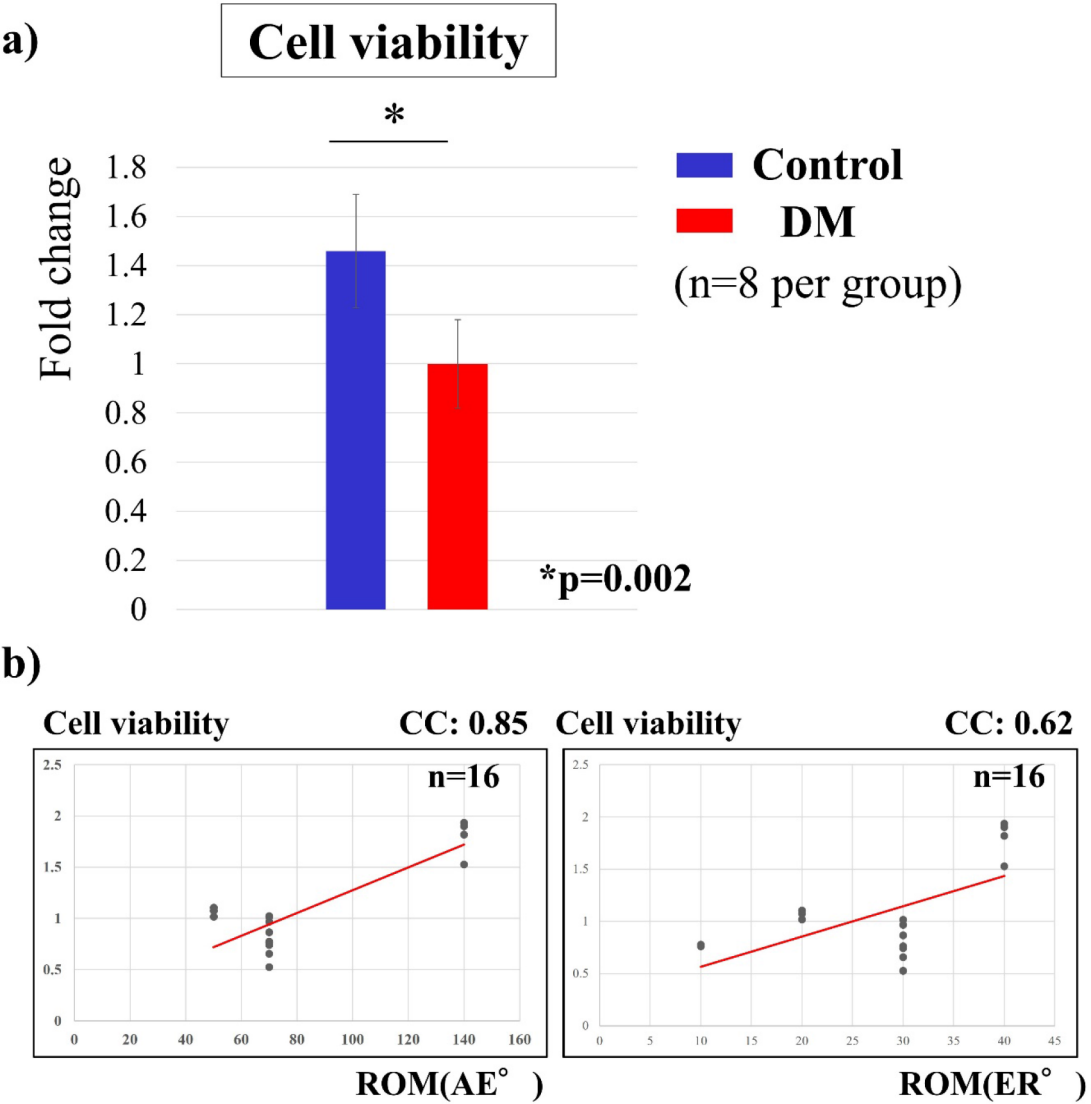
Viability of the shoulder capsule cells. **a** Cell viability was significantly lower in the DM group compared with that in the control group (*p* = 0.002). **b** There was a significant positive correlation between cell viability and shoulder joint ROM (*p* < 0.01)

Cell viability, expressed as fold change, was significantly lower in the DM group (1.46 ± 0.33 in the control group and 1.00 in the DM group, *p* = 0.002, Fig. 3a) than in the control group. In addition, there was a significant positive correlation between cell viability and shoulder joint ROM (AE: correlation coefficient [CC] = 0.85, *p* = 0.00004; ER: CC = 0.62, *p* = 0.01; Fig. 3b).

### ROS expression

**Fig. 4:**
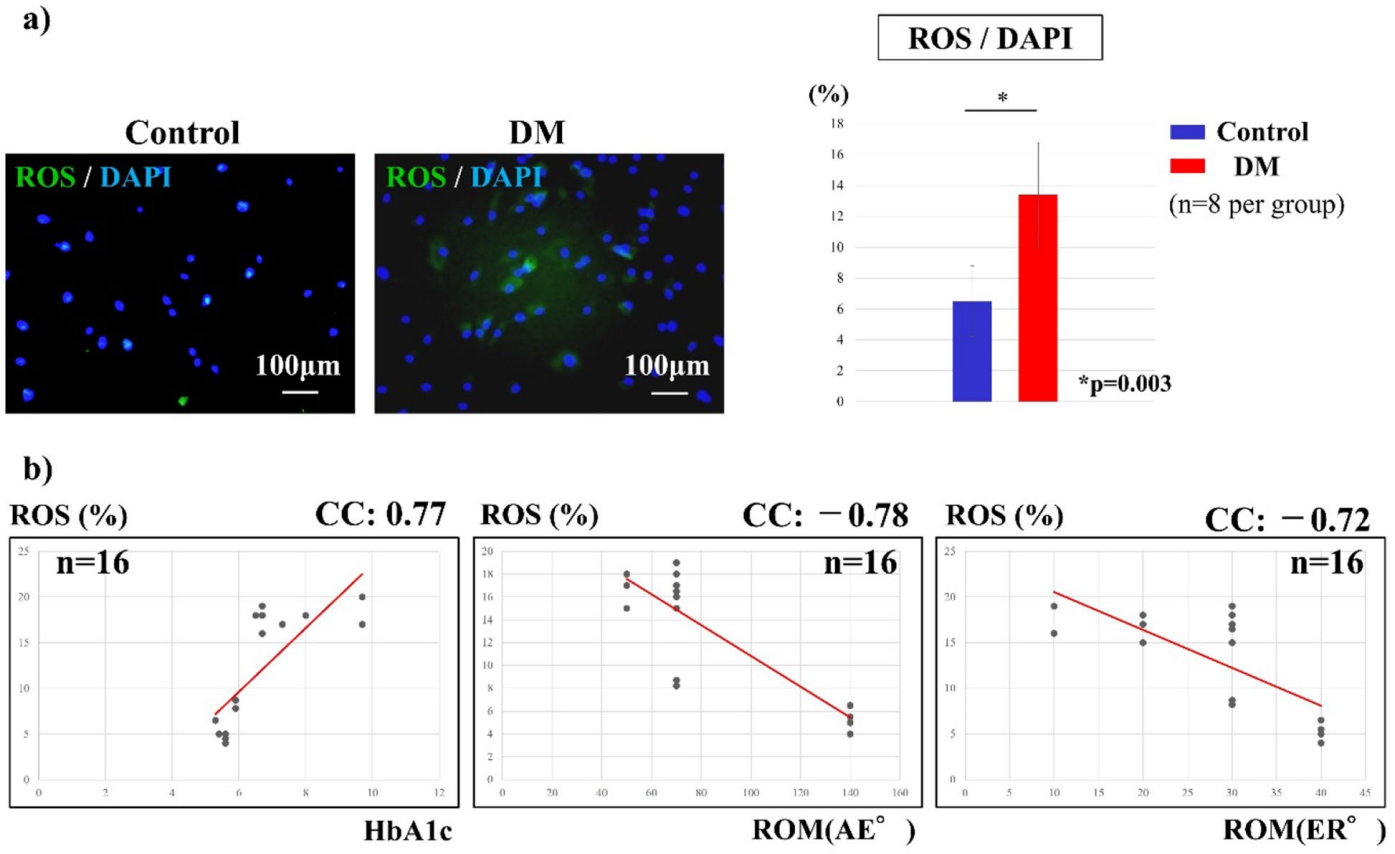
ROS staining of the shoulder capsule cells. **a** The percentage of ROS staining was significantly higher in the DM group (*p* = 0.003). **b** The percentage of ROS staining showed significant positive correlation with preoperative HbA1c (*p* < 0.01) and a significant negative correlation with the preoperative ROM of the shoulder joint (*p* < 0.01)

ROS staining was semi-quantified as the percentage of green-stained ROS to blue-stained DAPI cells. The staining percentage was 6.5 ± 1.8% in the control group and 13.4 ± 4.8% in the DM group, which was significantly higher in the DM group (*p* = 0.003, Fig. 4a). The percentage of ROS staining showed a significant correlation with the preoperative HbA1c level (CC = 0.77, *p* = 0.005; Fig. 4b). The percentage of ROS staining showed a significant negative correlation with the preoperative ROM of the shoulder joint (AE: CC = − 0.78, *p* = 0.0002; ER: CC = − 0.71, *p* = 0.002; Fig. 4b).

### Apoptosis rate

**Fig. 5:**
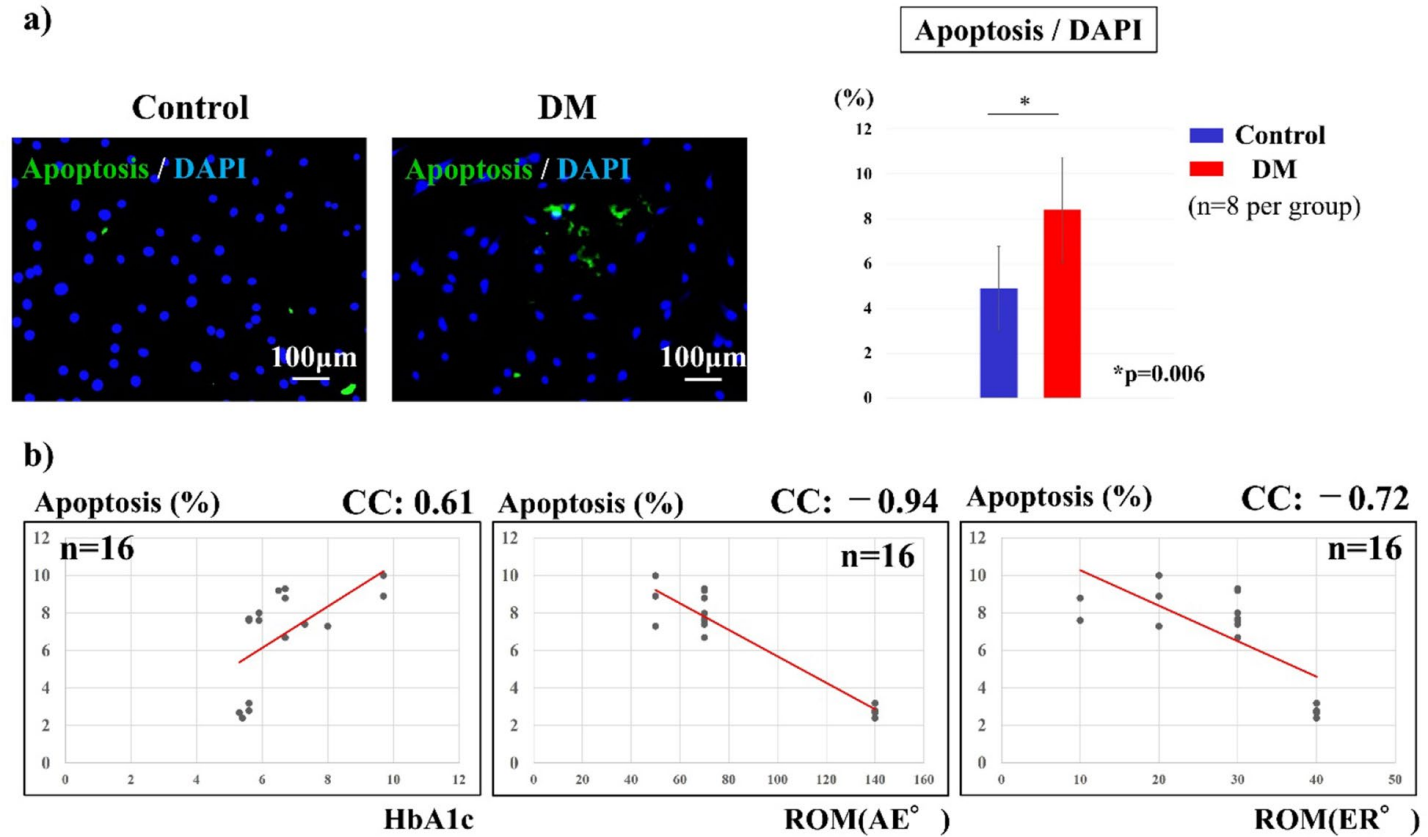
Apoptosis rate. a) The percentage of apoptosis was significantly higher in the DM group (*p* = 0.006). b) The percentage of apoptosis showed a significant positive correlation with preoperative HbA1c (*p* < 0.01) and a significant negative correlation with the preoperative ROM of the shoulder joint (*p* < 0.01)

The percentage of cells that underwent apoptosis was semi-quantified as the ratio of green-stained nuclear fragmentation to DAPI-stained cells. The percentage of apoptosis was 4.9 ± 2.4% in the control group and 8.4 ± 1.8% in the DM group, and was significantly higher in the DM group (*p* = 0.006, Fig. 5a). The percentage of apoptotic cells showed significant positive correlation with preoperative HbA1c (*p* = 0.01, CC = 0.61, Fig. 5b) and a significant negative correlation with the preoperative ROM of the shoulder joint (AE: CC = − 0.94, *p* = 0.001; ER: CC = − 0.72, *p* = 0.00009; Fig. 5b).

### Quantitative real-time PCR

**Fig. 6:**
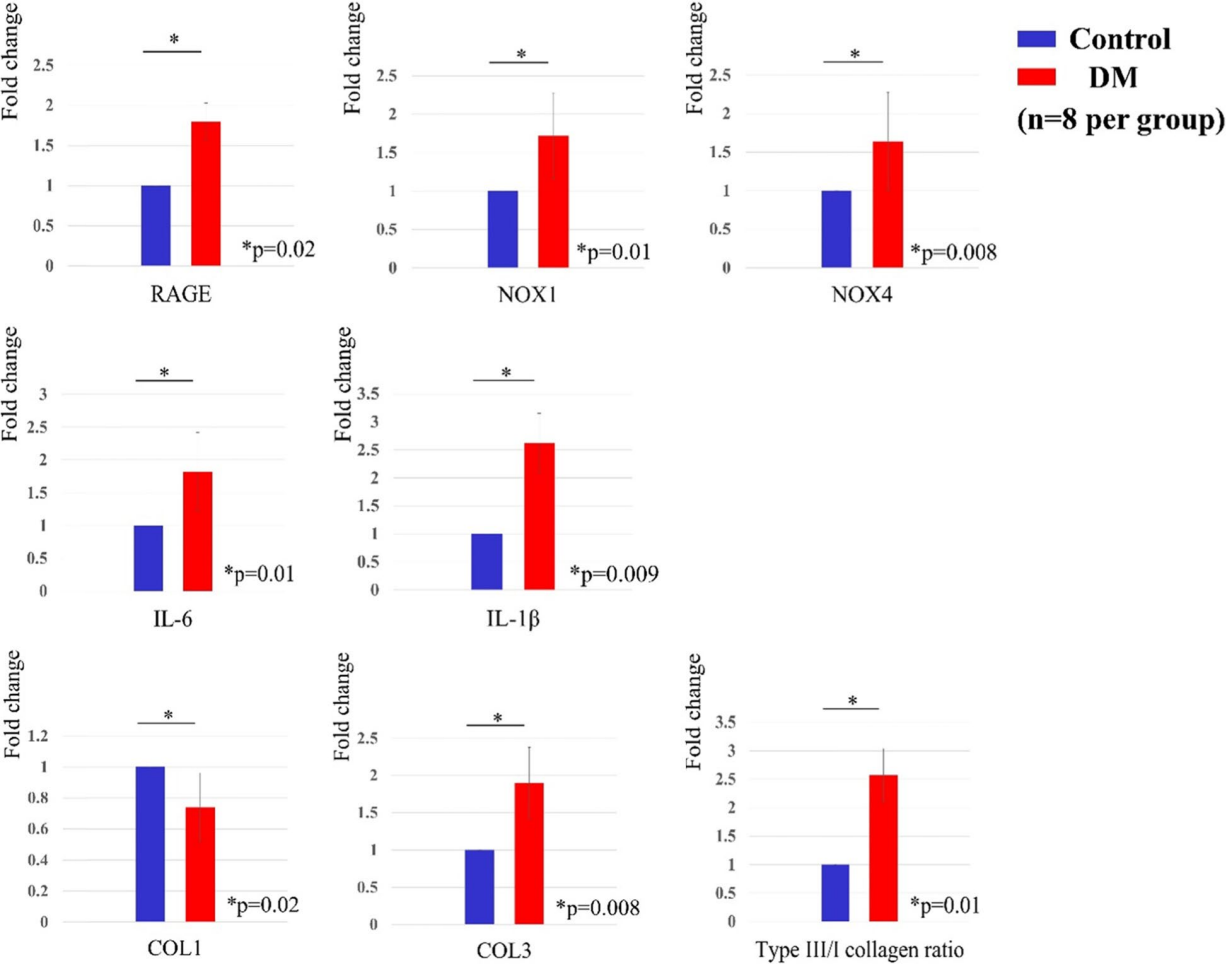
Results from real-time PCR

Gene expression was compared between the control and DM groups. The gene expression of NOX1, NOX4, and RAGE, which are involved in the increase of ROS, was significantly higher in the DM group (*p* = 0.01; NOX1, *p* = 0.008; NOX4, *p* = 0.02; RAGE, Fig. 6) than in the control group. The expression of inflammatory cytokines IL-6 and IL-1β was also significantly higher in the DM group (*p* = 0.01; IL-6, *p* = 0.009; IL-1β; Fig. 6) than in the control group. In terms of collagen expression, the COL1 and COL3 gene expression was significantly higher in the control group (*p* = 0.02) and the DM group (*p* = 0.008), respectively. The type III/I collagen ratio was significantly higher in the DM group (there was an increase by 1.00-fold and 2.57-fold in the control and DM groups, respectively. *p* = 0.01; Fig. 6) than in the control group.

## Discussion

In this study, we evaluated the effect of AGEs on the limited ROM of the shoulder with RCTs. AGEs were observed to accumulate in the shoulder capsule with limited ROM and was accompanied by increased oxidative stress and cell apoptosis. Increased oxidative stress and apoptosis were significantly correlated with HbA1c, an indicator of DM, and had a significant negative correlation with the ROM of the shoulder joint. Although previous reports have shown an association between DM and AGEs [12], this is the first report to demonstrate an association between limited ROM of the shoulder joint and AGEs. To our knowledge, this study is also the first to investigate the immunoreactivity of AGEs in the shoulder capsule in patients with RCTs displaying limited ROM.

AGEs are reported to cause aging in the skin, visceral organs, bone and cartilage, and lens, mainly because of the abnormal cross-linking of collagen [20]. Extracellular AGEs induce oxidative stress, inflammation, and apoptosis of cells in cardiovascular diseases and chronic kidney diseases [21, 22]. Additionally, research has shown that AGEs accumulate in collagen-rich long-lived tissues such as tendons and form intermolecular cross-links by bridging between the free amino groups of adjacent proteins, increasing tissue stiffness and brittleness [23]. Hwang et al. [6] reported that AGE accumulation in the shoulder capsule, which is rich in collagen [24], is higher in the frozen shoulder condition than in unstable shoulder or RCTs. The study has also shown that fibroblast proliferation and collagen fibrosis because of increased AGE expression are the causes of the frozen shoulder [6]. In this study, fibroblast proliferation was observed in the shoulder capsules with AGE accumulation. The ratio of type III to type I collagen (type III/I collagen ratio) is reported as an indicator of fibrosis [25]. It has been reported that the type III/I collagen ratio increases as fibrosis progresses, reflecting the proliferation of fibroblasts [26]. In tendon injuries, inflammatory cytokines such as IL-1β have been reported to significantly increase the levels of matrix metalloproteinases and decrease the expression of type I collagen, while increasing type III collagen mRNA levels in tendon cells, thereby contributing to tissue fibrosis [27]. In this study, the type III/I collagen ratio was significantly increased in the DM group, which had a higher accumulation of AGEs, suggesting that more fibrosis would occur. AGEs cause tissue fibrosis and inflammation by inducing oxidative stress in addition to the abnormal cross-linking of collagen [28, 29].

The binding of AGEs to RAGE activates NOX and increases ROS production, which is a source of oxidative stress [10]. Increased ROS levels lead to increased inflammatory cytokines owing to the activation of NFκB [28]. Increased ROS levels also induce mitochondrial DNA damage and activate caspases, thereby inducing apoptosis [29]. Increased levels of inflammatory cytokines and apoptosis result in fibrosis and tissue fragility [30]. In the present study, the increase in AGEs also resulted in increased gene expression of *RAGE* and *NOX* and increased ROS production. The increase in ROS levels was accompanied by an increase in apoptosis, a decrease in cellular viability, and an increase in inflammatory cytokines. As a result, fibrosis and adhesion to surrounding tissues can occur in the joint capsule, leading to limited ROM.

The risks of AGE accumulation include aging and DM [11, 31]. Hwang et al. reported that there was no significant difference in the accumulation of AGEs between patients with and without DM [6]. However, the number of DM patients studied was small (two cases), a major limitation. In the present study, AGE deposition in the shoulder capsule was higher in the DM group than in the control group. It was also suggested that the amount of AGEs deposited might affect the limitation of ROM of the shoulder joint. AGE deposition causes oxidative stress, leading to fibrosis and local inflammation, which can cause limited ROM of the shoulder joint. The treatment of AGEs could potentially prevent ROM limitation in RCT patients; however, further research is required to confirm this.

This study has several limitations. First, shoulder capsule tissues from patients without ROM limitation were not harvested owing to ethical reasons. Second, although a power analysis was performed, the sample size was not large. Further studies with more samples are required to confirm these results. Third, we did not compare the results according to the duration of DM. In addition, all patients in the DM group in this study had a preoperative HbA1c of 6.5 or higher, and the preventive effect of lowering the HbA1c to normal was not evaluated. HbA1c is considered an indicator of DM control, and its correlation with oxidative stress and apoptosis suggests that DM control could be important in preventing the ROM limitation. However, long-term prospective studies on the preventive effect of DM control are required. Finally, only DM has been examined as a contributing factor to limited ROM. The accumulation of AGEs may also be affected by age, and patient factors such as their daily schedules may also affect joint ROM. Further studies are required to confirm the results of this study.

## Conclusions

The DM group showed significant AGE deposition in the shoulder capsule. The findings of this study demonstrated a significant association between AGEs and ROM limitation. This study suggests that the oxidative stress induced by AGEs deposition, which leads to fibrosis and local inflammation, might contribute to the limited ROM of the shoulder joint in patients with RCTs accompanied by DM.

## Data Availability

The data presented in this study are available on request from the corresponding author. The data are not publicly available because of confidentiality issues.

## Abbreviations

RCT: Rotator cuff tear
ROM: Range of motion
DM: Diabetes mellitus
AGE: Advanced glycation end products
NOX: NADPH oxidase
ROS: Reactive oxygen species
RAGE: Receptor of advanced glycation end products
DMEM: Dulbecco’s modified Eagle’s medium
FBS: Fetal bovine serum
H&E: Hematoxylin and eosin
NIH: NATIONAL Institutes of Health
WST: Water-soluble tetrazolium
DCFH-DA: Dichloro-dihydro-fluorescein diacetate
DAPI: Diamidino-2-phenylindole
TUNEL: Terminal deoxynucleotidyl transferase dUTP nick end-labeling
AE: Anterior elevation
ER: External rotation
